# Meta-heuristic algorithms for parallel identical machines scheduling problem with weighted late work criterion and common due date

**DOI:** 10.1186/s40064-015-1559-5

**Published:** 2015-12-18

**Authors:** Zhenzhen Xu, Yongxing Zou, Xiangjie Kong

**Affiliations:** School of Software, Dalian University of Technology, Dalian, 116620 China

**Keywords:** Scheduling, Parallel identical machines, Common due date, Meta-heuristic algorithms

## Abstract

To our knowledge, this paper investigates the first application of meta-heuristic algorithms to tackle the parallel machines scheduling problem with weighted late work criterion and common due date ($P|{{d}_{j}}=d|{{Y}_{w}}$). Late work criterion is one of the performance measures of scheduling problems which considers the length of late parts of particular jobs when evaluating the quality of scheduling. Since this problem is known to be NP-hard, three meta-heuristic algorithms, namely ant colony system, genetic algorithm, and simulated annealing are designed and implemented, respectively. We also propose a novel algorithm named LDF (largest density first) which is improved from LPT (longest processing time first). The computational experiments compared these meta-heuristic algorithms with LDF, LPT and LS (list scheduling), and the experimental results show that SA performs the best in most cases. However, LDF is better than SA in some conditions, moreover, the running time of LDF is much shorter than SA.

## Background

Time constrain condition is widely employed in various real-life problems, which can be used to determine the feasibility conditions and makes it possible to estimate the quality of feasible solution corresponding to those problems. In scheduling theory, we usually model time restrictions by due dates and deadlines, and we evaluate the quality of scheduling by taking into account these parameters. Researchers have proposed several performance criterions base on these models, such as maximum lateness (McMahon and Florian 1975), total tardiness (Lawler 1977), mean tardiness (Kim and Yano 1994), and the number of tardy jobs (Moore 1968; Cheng et al. 2006). Most research literatures always focus on these classical performance measures, while the late work criterion has not been so widely studied.

The late work concept was first proposed in the context of an identical parallel machines scheduling problem by Blazewicz (1984), who called it “information loss”. The phrase “late work” was first proposed by Potts and Van Wassenhove (1992), who applied it to the single machine cases. Other researchers such as Hochbaum and Shamir (1990), Hariri et al. (1995), Kovalyov and Kubiak (1999), Kethley and Alidaee (2002) also studied the single machine cases. Then, the late work performance measures were applied to the shop scheduling problems (Błażewicz et al. 2000). Similarly, Blazewicz et al. (2004) focused on the open shop scheduling problem and Blazewicz et al. (2005b) studied the two-machine flow-shop problem. A number of scholars used meta-heuristic approaches to solve the scheduling problem with late work criterion in dedicated machine case. In Blazewicz et al. (2005a) and (2008), three meta-heuristic approaches (tabu search, simulated annealing and variable neighborhood search) were applied to the $F2|{{d}_{j}}=d|{{Y}_{w}}$ problem, and genetic algorithm (GA) was used to solve the problem of $F|{{r}_{j}}|Y$ (Sterna et al. 2007).

However, meta-heuristic algorithms have not been used in the context of parallel machines case. We propose, for the first time, three meta-heuristic algorithms for the parallel identical machines scheduling problem with weighted late work criterion and common due date which is denoted by $P|{{d}_{j}}=d|{{Y}_{w}}$. Three meta-heuristic algorithms include ant colony system algorithm (ACS), genetic algorithm (GA), and simulated annealing (SA).

Two typical scheduling algorithms were also implemented: longest processing time first (LPT) and list scheduling (LS). The LPT should sort all jobs based on their processing time first, and assign each job to a machine with minimum load. While in LS, a job will be directly assigned to the machine with minimum load. Additionally, we proposed a novel algorithm named LDF (largest density first) which is an improved algorithm of LPT. In the simulation experiments, six algorithms mentioned above were compared with each other under different values of parameters.

The rest of the paper is organized as follows. The problem statement of $P|{{d}_{j}}=d|{{Y}_{w}}$ is introduced in “Problem statement” section. The design and application of meta-heuristic algorithms for $P|{{d}_{j}}=d|{{Y}_{w}}$ are described in detail in “[Sec Sec3]” section. “Largest density first algorithm” section gives a new method which is improved from LPT. “Computational experiments” section analyses the computational experiment results performed by these algorithms. In “Conclusions” section, some conclusions are given.

## Problem statement

Generally, the scheduling problem can be defined as assigning a set of jobs to a set of machines under given constrained conditions (Sterna 2011). Hence, the parallel identical machines scheduling problem with weighted late work criterion, could be defined as follows. Given *n* jobs $J=\{{{J}_{1}},{{J}_{2}},\ldots ,{{J}_{j}},\ldots ,{{J}_{n}}\}$ and *m* identical machines $M=\{M_{1},M_{2},\ldots ,M_{i},\ldots ,M_{m}\}$, each job ${{J}_{j}}\ (j=1,2,\ldots ,n)$ is mainly described by its processing time ${{p}_{j}}$, due date ${{d}_{j}}$ and weight ${{w}_{j}}$. Let ${{d}_{j}}$ represents the preferred completion time for this job, and weight ${{w}_{j}}$ represents the relative importance of this job. Each job can be executed on one of the machines and each machine can execute only one job at a time. In this paper, we focus on a common due date *d* for all jobs (i.e. ${d}_{j}=d$) and we look for a non-preemption schedule that minimizes the total weighted late work ${{Y}_{w}}.$

**Fig. 1:**
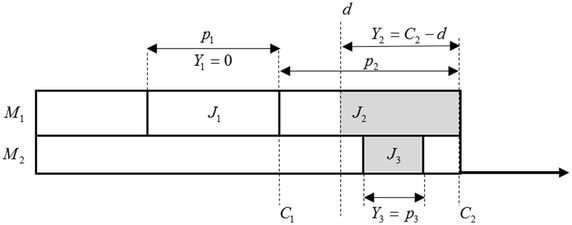
Late work definition schematic diagram. In this figure, *d* is the common due date, ${{p}_{j}}$ denotes the processing time of each job ${{J}_{j}}\ (j=1,2,\ldots ,n)$, and ${{C}_{j}}$ denotes the completion time of job ${{J}_{j}}$, then the late work ${{Y}_{j}}$ for this job is the minimum of ${{p}_{j}}$ and $max\{0,{C}_{j}-d\}$

The completion time of job ${{J}_{j}}$ is denoted by ${{C}_{j}}$, the late work ${{Y}_{j}}$ for this job is given by the following formula (cf. Fig. 1):

$$\begin{aligned} {{Y}_{j}}=\min \{{{p}_{j}},\max \{0,{{C}_{j}}-d\}\} \end{aligned}$$

According to this definition, we can conclude that the late work criterion evaluates the quality of a feasible solution based on the length of late parts of particular jobs. Late work concentrates the advantages of two kinds of parameters: tardiness and the number of tardy jobs.

The total weighted late work can be defined as the weighted sum of late works of all jobs, is given by the following formula:

$$\begin{aligned} {{Y}_{w}}=\sum \limits _{j=1}^{n}{{{w}_{j}}{{Y}_{j}}}=\sum \limits _{j=1}^{n}{{{w}_{j}}\min \{{{p}_{j}},\max \{0,{{C}_{j}}-d\}\}} \end{aligned}$$

## Meta-heuristic algorithms for $\varvec{P|{{d}_{j}}=d|{{Y}_{w}}}$

In this section, we design three meta-heuristic algorithms to solve the problem $P|{{d}_{j}}=d|{{Y}_{w}}$ , respectively. The parameters setting of these algorithms will be given in “Computational experiments” section.

### ACS for $P|{{d}_{j}}=d|{{Y}_{w}}$

ACS algorithm is one of the successful variations of ant colony optimization (ACO) (Dorigo and Gambardella 1997). Recently, ACO methods have been widely used in various scheduling problems, such as single machine case considered by Merkle and Middendorf (2003) and Bauer et al. (1999), job-shop problem (Dorigo et al. 1996; Colorni et al. 1994) and flow-shop case (Stutzle 1998). These research literatures show that ACO methods perform much better than other heuristic algorithms in finding the optimal solution of some benchmark problems (Merkle and Middendorf 2003).

#### Definition of heuristic information

When assigning a job, an ant should first choose a machine ${{M}_{i}}$ as the processing machine randomly, and then add a job ${{J}_{j}}$ to the scheduling sequence of machine ${{M}_{i}}$ based on heuristic information and pheromone. The heuristic information is denoted by ${{\eta }_{i}}_{j}$, which indicates the expectation of selecting job ${{J}_{j}}$ to assign to machine ${{M}_{i}}$. The value of ${{\eta }_{i}}_{j}$ is calculated according to the heuristic rule named variation of modified due date rule (VMDD) (Merkle and Middendorf 2003), i.e.,

$$\begin{aligned} {{\eta }_{ij}}=\frac{{{w}_{j}}}{\max \left\{ {{T}_{i}}+{{p}_{j}},d \right\} -{{T}_{i}}} \end{aligned}$$

where ${{T}_{i}}$ indicates the total processing time of all jobs which are already assigned to machine ${{M}_{i}}$.

#### Pheromone initialization

Similar to the definition of heuristic information, the pheromone is denoted by ${{\tau }_{ij}}$, indicates the pheromone value of choosing a job ${{J}_{j}}$ to assign to machine ${{M}_{i}}$. We initialize the pheromone value as ${{\tau }_{0}}=1/n{{L}_{H}}$, where ${{L}_{H}}$ is the objective function value obtained by the initial solution.

#### Solution construction and pheromone updating

Initially, the algorithm generates an initial solution randomly and obtains the initial pheromone value ${{\tau }_{0}}$. Each ant should finish the solution construction process and pheromone updating process. An ant *k* located on machine ${{M}_{i}}$ chooses a next job ${{J}_{j}}$ based on the pseudo random proportional rule, i.e.,

$$j = \left\{ {\begin{array}{*{20}l} {\mathop {arg\max }\limits_{{l \in N_{k} }} \left\{ {\tau _{{il}} [\eta _{{ij}} ]^{\beta } } \right\}} \hfill & \quad {{\text{if}}\;q \le q_{0} } \hfill \\ {J,} \hfill & \quad {{\text{otherwise}}} \end{array} } \right.$$

If $q>{{q}_{0}}$, the probability of choosing job ${{J}_{j}}$ as the next job could be formulated as follows:

$$p_{{ij}}^{k} = \left\{ {\begin{array}{*{20}l} {\frac{{[\tau _{{ij}} ]^{\alpha } [\eta _{{ij}} ]^{\beta } }}{{\sum\limits_{{l \in N_{k} }} {[\tau _{{il}} ]^{\alpha } [\eta _{{il}} ]^{\beta } } }},} \hfill & \quad {{\text{if}}\;j \in N_{i}^{k} } \hfill \\ {0,} \hfill & \quad {{\text{otherwise}}} \end{array} } \right.$$

where *q* is a random uniform variable in [0,1], and ${{q}_{0}}\ (0\le {{q}_{0}}\le 1)$ is a parameter to determine the relative importance between exploitation of a priori knowledge and exploration of a new edge. $\alpha$ and $\beta$ are factors whose value determines the relative influence of pheromone and heuristic information, respectively. ${{N}_{k}}$ is the set of jobs that have not been visited by ant *k* so far.

To avoid premature convergence, an ant should conduct local pheromone updating after selecting a job ${{J}_{j}}$, according to the following rule:

$$\begin{aligned} {{\tau }_{ij}}=(1-\xi ){{\tau }_{ij}}+\xi {{\tau }_{0}} \end{aligned}$$

where $\xi (0<\xi <1)$ represents the local pheromone evaporation rate. After all of the ants have constructed their valid solutions, the pheromone of the best solution found so far should be updated according to the following rule:

$$\begin{aligned} {{\tau }_{ij}}=(1-\rho ){{\tau }_{ij}}+\rho \Delta \tau _{ij}^{best}, \quad {\text {if}}\ (i,j)\in {{S}^{best}} \end{aligned}$$

where ${{S}^{best}}$ represents the best solution found so far and $\Delta \tau _{ij}^{best}$ denotes the inverse of the objective function value obtained by ${{S}^{best}}$. $\rho$ is used to control the global pheromone evaporation rate. The algorithm repeats these steps above until meeting the terminal condition, then the ${{S}^{best}}$ will be the ultimate solution obtained by ACS. In this paper, we set the terminal condition as the maximum number of iteration.

### GA for $P|{{d}_{j}}=d|{{Y}_{w}}$

The genetic algorithm (GA) is an optimization technique inspired by natural evolution, and it is widely used in various realistic scheduling problem such as berth allocation (Pratap et al. 2015). In the GA, the individuals (called chromosomes) in a population are encoded to present candidate solutions to an optimization problem. The evolution usually starts with an initial population generated randomly, then the fitness of each individual in the population will be evaluated and a selection mechanism is applied according to the fitness. Then the crossover and mutation operators are applied to generate the offspring. The algorithm repeats these steps until reach the maximum number of iteration.

#### Chromosomes encoding

In this paper, we adopt a two-dimensional encoding method. Each gene is constituted by a tuple: $({{x}_{j}},{{y}_{j}})$, $1\le j\le n$, where ${{x}_{j}}$ denotes the number of the machine which job ${{J}_{j}}$ is assigned to, and ${{y}_{j}}$ represents that ${{J}_{j}}$ is the ${{y}_{j}}\text {-th}$ job in the job sequence on the current machine. For example, given five jobs and two machines, then we can construct a chromosome with five genes to represent a candidate solution: (1,2), (2,1), (2,2), (1,3), (1,1). The above chromosome means job ${{J}_{1}}$ is assigned to ${{m}_{1}}$ and it is the second job on that machine, and ${{J}_{2}}$ is assigned to ${{m}_{2}}$ and it is the first job to be executed on that machine, and so on.

#### Initial population and fitness function

The initial population is generated randomly. The fitness function evaluates the quality of an individual in the population. Since it is a minimization problem, the fitness function can be designed as follows:

$$\begin{aligned} f(i)=M-{{Y}_{w}}(i) \end{aligned}$$

where ${{Y}_{w}}(i)$ is the objective function value (weighted late work) obtained by individual *i*, and *M* is a constant which guarantees that the fitness value is positive.

#### Selection

In this algorithm, the selection operator is based on roulette rule. The probability of selecting the *i*-th individual can be defined as:

$$\begin{aligned} {{p}_{i}}=\frac{f(i)}{\sum \nolimits _{k=1}^{popsize}{f(k)}} \end{aligned}$$

where *popsize* is the population size, and *f*(*i*) denotes the fitness value of the *i*-th individual.

#### Crossover

In order to generate the offspring, information between two selected parents should be exchanged. In this paper, we consider the single point crossover method. Assuming that the crossover rate is ${{p}_{c}}$ , a crossover process can be described as follows:

1. Generate a random number $p\in (0,1)$, if $p>{{p}_{c}}$ , then go to (2), else exit this process.
2. Generate a random integer number $l\in [1,n]$ , where *n* is the number of all jobs, also the length of a chromosome. Then, exchange the genes from *l* to *n* between two selected parents. Go to (3).
3. Repeat (1) and (2) for all adjacent chromosomes.

#### Mutation

Mutation operator can help avoid getting trapped in local optimum. In this process, the procedure traverses all individuals in a population, for each individual, the algorithm judges whether it should perform the mutation operator according to the mutation rate ${{p}_{m}}$. When an individual is selected to mutate, we randomly pick a job from it, and reassign this job to another random position which can be on the current machine or on other machines.

### SA for $P|{{d}_{j}}=d|{{Y}_{w}}$

Simulated annealing (SA) is a random optimization algorithm based on Mente Carlo simulation, which was inspired by the similarity between the annealing process of solid matters and combinational optimization problems. The algorithm was widely applied to the combinational optimization problems (Kirkpatrick et al. 1983; Van Laarhoven and Aarts 1987; Hazir et al. 2008).

#### Initialization and termination condition

In this paper, we generate an initial solution randomly with an initial temperature. The algorithm is terminated after reaching a minimum temperature or exceeding a given number of iterations.

#### Neighborhood structure

In order to explore the solution space, two operators are given to generate a neighbor: job move (${{N}_{1}}$) and jobs interchange (${{N}_{2}}$).

In the job move operator, a neighbor is generated by selecting a job from a current solution randomly and moving it to another random position. Jobs interchange operator generates a new neighbor through swapping two jobs selected randomly from different machines. The SA selects an operator randomly at each iteration, and performs it $\left\lfloor m/2 \right\rfloor$ times, and then obtain a new neighbor.

#### Cooling scheme

In this paper, we consider the geometric cooling scheme, which was a typical scheme that widely used in many scheduling problems (Hasani et al. 2014). In this scheme, the temperature is updated according to:

$$\begin{aligned} {{T}_{k}}=\theta {{T}_{k-1}},\quad k=1,2,\ldots \end{aligned}$$

where $0<\theta <1$ is the cooling factor. ${{T}_{k}}$ is reduced after running ${{I}_{iter}}$ iterations.

#### Proposed algorithm procedure

The pseudo-code of the proposed algorithm is described as follows: 

## Largest density first algorithm

We propose a novel algorithm named LDF (largest density first). This algorithm is improved from LPT (longest processing time first). Considered the weight of each job, we define the density of a job ${{J}_{j}}$ as ${{w}_{j}}/{{p}_{j}}$. The main idea of this algorithm is assigning the jobs to machines based on their density. The job with the largest density will be scheduled first and assigned to the machine with minimum load. The pseudo-code of LDF is described as follows: 

## Computational experiments

### Parameters setting

In the ACS algorithm, parameters are set as: ${{q}_{0}}=0.8$, $\alpha =1.0$, $\beta =3.0$, $\rho =0.5$, $\xi =0.1$. Set the size of ant colony $AntSize=30$ , and the maximum number of iteration ${{I}_{\max }}=50$.

The parameters setting of GA are as follows: set the size of population $PopSize=30$, the maximum number of iteration ${{G}_{\max }}=300,$ and ${{p}_{c}}=0.8$, ${{p}_{m}}=0.01$.

In the SA, set ${{T}_{0}}\text {=5} \cdot \sum \nolimits _{i=1}^{n}{{{p}_{i}}}$, $\theta = 0.975$, and ${{I}_{iter}}=20$.

Note that a solution with ${{Y}_{w}} = 0$ must be an optimal solution, so the algorithms will “early exit” when generate a solution with ${{Y}_{w}}\text {=}0$.

In order to simulate different experimental cases, we also need to set five input parameters, which are the number of machines *m*, the number of jobs *n*, the processing time of each job *p*, the weight of each job *w*, and the common due date *d*. The settings of these input parameters are shown in Table 1.

**Table 1:** Settings of five input parameters

*m*	*n*	*p*	*w*	*d*
		*U*[1,10*n*]	*U*[1, 3*m*]	$\left\lfloor \mu \cdot \frac{1}{m}\sum \nolimits _{i=1}^{n}{{{p}_{i}}} \right\rfloor$
		*U*[1,20*n*]	*U*[1, 5*m*]	
{2, 3, 5, 10}	{3*m*, 5*m*, 10*m*, 15*m*}	Poisson distribution where		
		$\lambda =500$	*U*[1, 50]	
		*U*[100 − 5,100 + 5]	*U*[1, 100]	

The value of *p* is an integer and it will be rounded down in Poisson distribution. $\mu$ is a parameter that belongs to the set ${\text {0.8}, \text {0.9}, \text {1.1}, \text {1.2}}$, which controls the value of *d*. For each group of the five input parameters, 20 different experimental instances are generated to run each algorithm 20 times. For example, set $m=2$ and $n=3m$ (i.e. $n=6$), assume *p* is generated randomly from *U*[1,10n] (i.e. *U*[1,60]), *w* is generated from *U*[1,100], set $\mu =0.8$ and we can obtain the corresponding *d*. Then, 20 different experimental instances are generated according to this group of parameters. The experiments are conducted on a server with win server operating system, E5-2407 CPU and 32G memory.

### Results and analysis

**Fig. 2:**
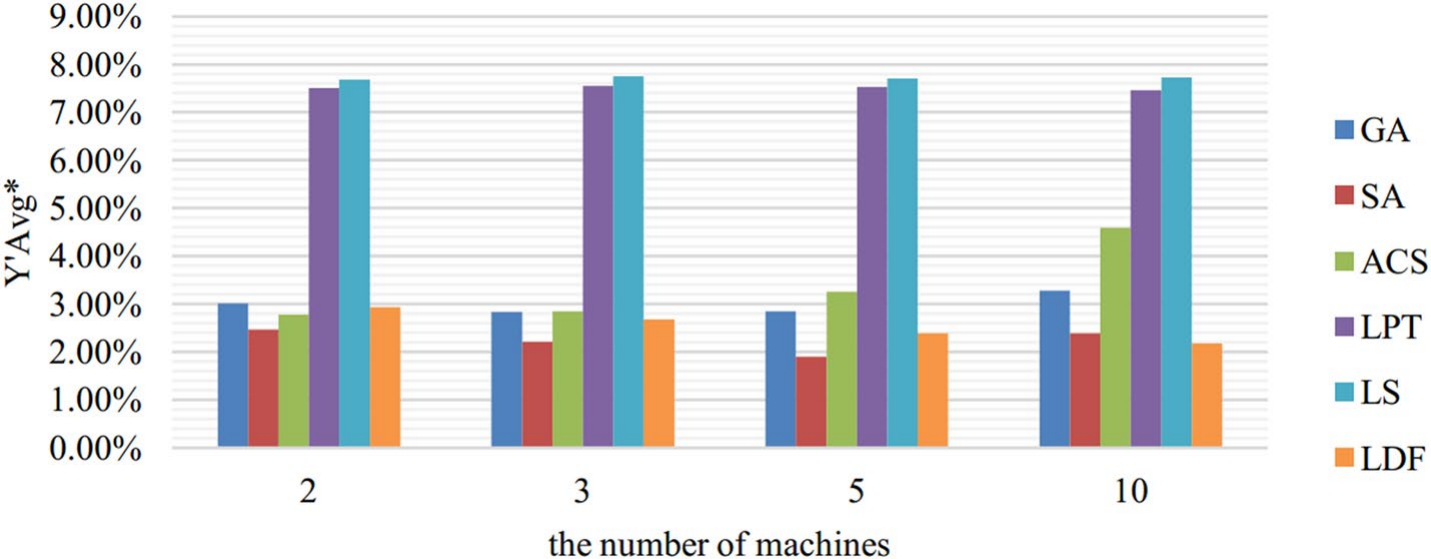
Comparison results in ${Y}'Avg^*$ merged by *m*. This figure shows the comparison results of six algorithms in ${Y}'Avg^*$ which are merged by ${Y}'Avg$ with the same *m*. ${Y}'Avg$ is denoted as the average value of ${{Y}_{w}}/\sum \nolimits _{i=1}^{n}{{{p}_{i}}{{w}_{i}}}$

**Fig. 3:**
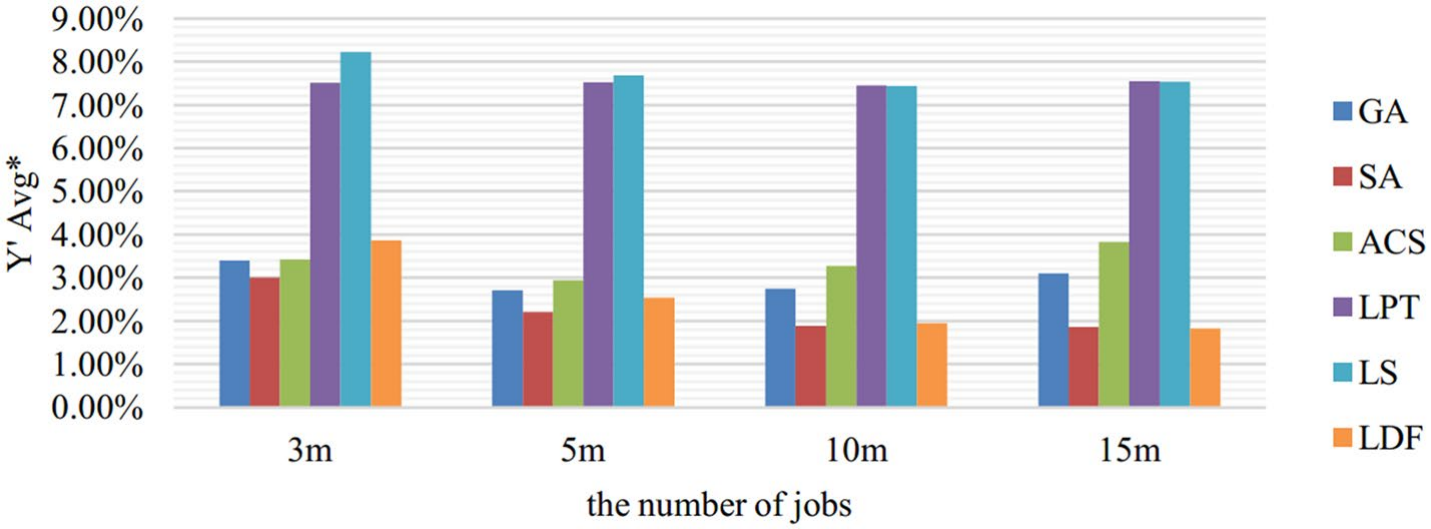
Comparison results in ${Y}'Avg^*$ merged by *n*. This figure shows the comparison results of six algorithms in ${Y}'Avg^*$ which are merged by ${Y}'Avg$ with the same *n*. ${Y}'Avg$ is denoted as the average value of ${{Y}_{w}}/\sum \nolimits _{i=1}^{n}{{{p}_{i}}{{w}_{i}}}$

**Fig. 4:**
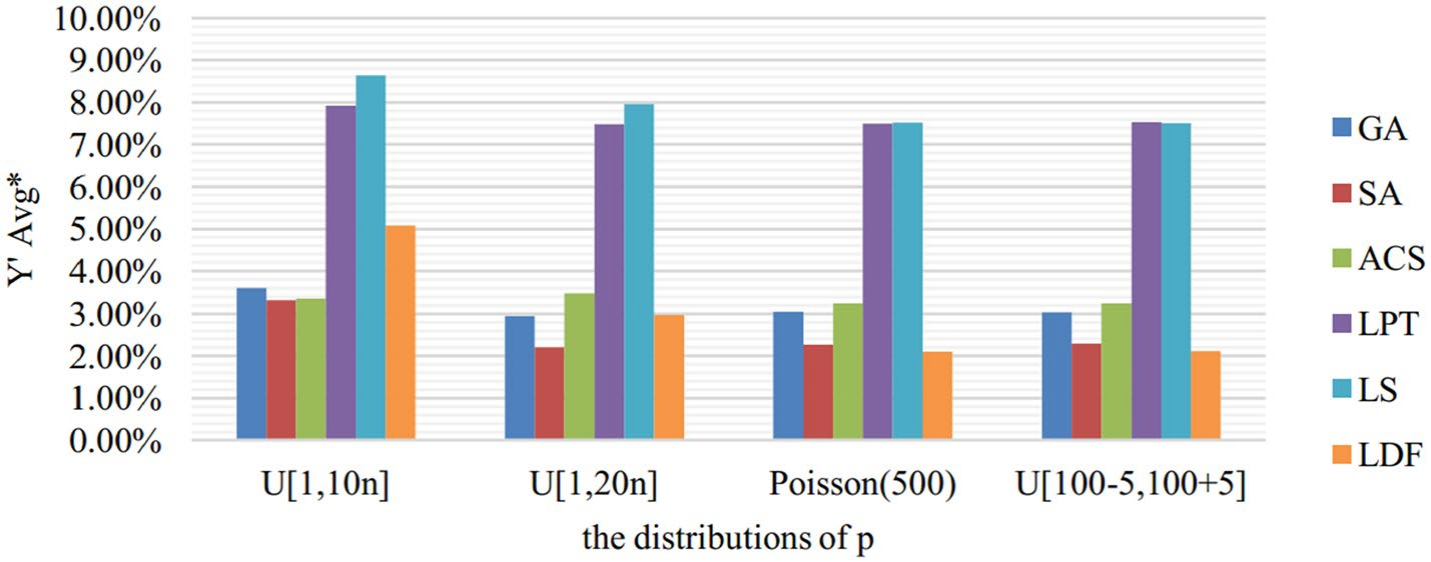
Comparison results in ${Y}'Avg^*$ merged by *p*. This figure shows the comparison results of six algorithms in ${Y}'Avg^*$ which are merged by ${Y}'Avg$ with the same *p*. ${Y}'Avg$ is denoted as the average value of ${{Y}_{w}}/\sum \nolimits _{i=1}^{n}{{{p}_{i}}{{w}_{i}}}$

**Fig. 5:**
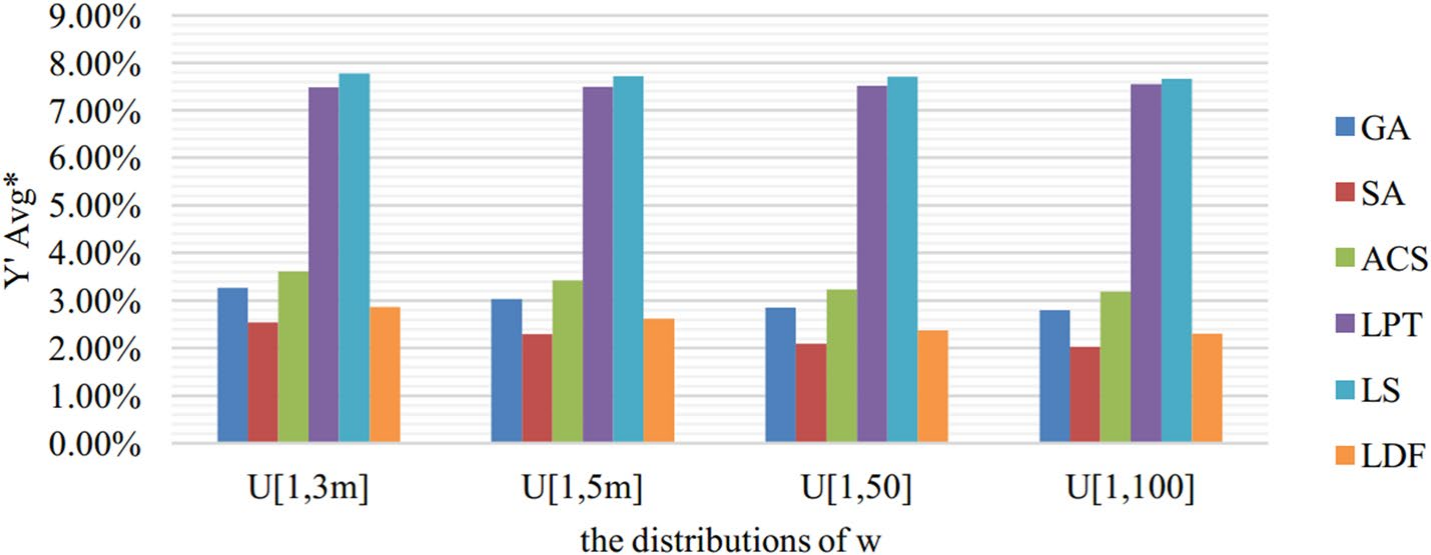
Comparison results in ${Y}'Avg^*$ merged by *w*. This figure shows the comparison results of six algorithms in ${Y}'Avg^*$ which are merged by ${Y}'Avg$ with the same *w*. ${Y}'Avg$ is denoted as the average value of ${{Y}_{w}}/\sum \nolimits _{i=1}^{n}{{{p}_{i}}{{w}_{i}}}$

**Fig. 6:**
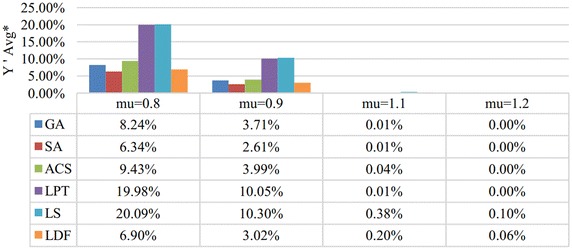
Comparison results in ${Y}'Avg^*$ merged by $\mu$. This figure shows the comparison results of six algorithms in ${Y}'Avg^*$ which are merged by ${Y}'Avg$ with the same $\mu$. ${Y}'Avg$ is denoted as the average value of ${{Y}_{w}}/\sum \nolimits _{i=1}^{n}{{{p}_{i}}{{w}_{i}}}$

For each group of experiments, six different algorithms are all carried out. Because the orders of magnitudes of ${{Y}_{w}}$ are quite different under different parameter values, we use ${{Y}_{w}}/\sum \nolimits _{i=1}^{n}{{{p}_{i}}{{w}_{i}}}$ to evaluate and compare the quality of a solution. The average values of ${{Y}_{w}}/\sum \nolimits _{i=1}^{n}{{{p}_{i}}{{w}_{i}}}$ (in percent, denoted by ${Y}'Avg$) and the average running time *TimeAvg* (in seconds) of every 20 test instances are computed. Figs. 2, 3, 4, 5 and 6 show the comparison results in ${Y}'Avg^*$, which are merged by ${Y}'Avg$ with the same *m*, *n*, *p*, *w* and $\mu$, respectively, due to the space limitations.

As we can see from Figs. 2 and 3, SA performs best in most cases, and LDF is slightly better than SA when the problem scale is large, i.e., $m=10$ or $n=15m$. In Fig. 2, the performance of ACS is significantly influenced by the parameter *m* which related to the problem scale. ACS is getting worse and worse with the increasing number of machines. Fig. 3 shows that LDF is greatly influenced by the parameter *n*. LDF is worse than three meta-heuristic algorithms when $n=3m$ but better than ACS and GA with the increasing ratio from 5 to 15.

In Fig. 4, we can see that the distributions of *p* have a greater impact on LDF compared with others. LDF is much worse than SA when *p* obeys *U*[1,10n] or *U*[1,20n]. However, it outperforms SA when *p* obeys Poisson distribution or *U*[100−5,100+5]. That means LDF can get better performance when the job procession time is concentrated.

The distributions of *w* gives no evident influence on all these algorithms and SA performs the best in all cases according to Fig. 5. Figure 6 shows the comparison results merged by different common due dates. When $\mu =1.1$ and $\mu =1.2$, almost all of these algorithms can obtain the optimal solutions ($Y'Avg=0\,\%$), and LDF is a little worse than three meta-heuristic algorithms. When $\mu =0.8$ and $\mu =0.9$, the performance of proposed algorithms from high to low are SA, LDF, GA and ACS.

According to Figs. 2, 3, 4, 5 and 6, we can conclude that SA performs best in most cases. LDF also shows excellent performances and it is even better than SA in certain conditions. Though GA and ACS are not so good, they are greatly better than LPT and LS.

The running times of these algorithms are mainly affected by the scale of problem. Note that in meta-heuristic algorithms, we adopt an “early exit” mechanism proposed above when the algorithms find a solution with ${{Y}_{w}}=0$. Hence in $\mu =1.1$ and $\mu =1.2$, the meta-heuristic run significantly faster than in other situations. Tables 2,  3 and 4 give the values of $TimeAvg^*$ which merged the *TimeAvg* by *m*, *n* and $\mu$, respectively.

**Table 2:** Comparison results in $TimeAvg^*$ by *m*

*m*	$TimeAvg^*$					
	GA	SA	ACS	LPT	LS	LDF
2	1.1111	0.5938	1.8501	0.0005	0.0005	0.0005
3	1.6686	0.7714	4.9555	0.0008	0.0007	0.0008
5	3.5004	1.7310	24.6330	0.0013	0.0014	0.0017
10	7.0229	4.9816	58.5864	0.0030	0.0028	0.0031

**Table 3:** Comparison results in $TimeAvg^*$ by *n*

*n*	$TimeAvg^*$					
	GA	SA	ACS	LPT	LS	LDF
3*m*	1.5580	1.4967	7.1742	0.0005	0.0005	0.0005
5*m*	2.3343	1.7119	13.4007	0.0009	0.0008	0.0011
10*m*	3.8761	2.0118	27.4117	0.0016	0.0015	0.0016
15*m*	5.5346	2.8575	42.0384	0.0027	0.0026	0.0029

**Table 4:** Comparison results in $TimeAvg^*$ by $\mu$

$\mu$	$TimeAvg^*$					
	GA	SA	ACS	LPT	LS	LDF
0.8	6.1848	3.1833	39.1215	0.0014	0.0014	0.0015
0.9	6.2162	3.1991	39.5560	0.0014	0.0013	0.0015
1.1	0.7111	1.0418	9.8085	0.0014	0.0014	0.0015
1.2	0.1909	0.6536	1.5389	0.0014	0.0014	0.0016

The average running time of GA and SA are much shorter than ACS in large scale problems according to Tables 2 and 3. The running time of LPT, LS and LDF are quite small even in large scale problem. In Table 4, the values of meta-heuristic algorithms in $\mu = 0.8$ and $\mu =0.9$ present the average running time merged by $\mu$ without “early exit” mechanism and SA is the best.

To make a comparison among the meta-heuristic algorithms in detail, the average number of better solutions is counted. The comparison results of meta-heuristic algorithms in the percent of better solutions are shown in Table 5, which is merged by $\mu$.

**Table 5:** Comparisons of meta-heuristic algorithms in the percent of better solutions

$\mu$	ACS	vs	GA	ACS	vs	SA	GA	vs	SA
0.8	6.32		12.64	1.06		16.48	1.27		17.54
0.9	8.89		9.84	1.70		15.33	1.63		16.99
1.1	0.13		1.77	0.42		1.74	0.69		0.41
1.2	0.02		0.16	0.20		0.17	0.20		0.03

The first column of Table 5 presents the values of $\mu$. The rest columns are divided into three groups. In each group, two meta-heuristic algorithms are compared. The first column and the second column in each group show the average amount of better solutions obtained by these two algorithms, respectively. For example, the value “12.64” at the first row and the third column means that when $\mu =0.8$ and traversing all other parameters, the GA generated an average number of 12.64 solutions which are better than that of ACS. At the same situation, the number of solutions which have equal quality in ACS and GA can be calculated by 20 − 6.32 − 12.64 = 1.04.

As we can see from Table 5, SA can usually generate better solutions, apart from the solutions with equal quality to other algorithms.

Combining the above analysis, we can conclude that SA performs best in most cases while its time cost is also acceptable. ACS and GA are also much better than the typical scheduling algorithms LPT and LS, but they have no advantages in comparison with LDF in most cases.

## Conclusions

In this paper, we proposed three meta-heuristic algorithms (i.e., ACS, GA and SA) and a novel LDF algorithm to solve the scheduling problem $P|{{d}_{j}}=d|{{Y}_{w}}$ for the first time. The proposed algorithms were compared to two typical scheduling algorithms LPT and LS. The computational experiments demonstrated that SA performed best in terms of finding optimal solutions compared to others in most cases, while the runtime of SA was acceptable in practical problems. The quality of solutions obtained by LDF is better than SA when the problem scale is large or the job processing time is concentrated. LDF also have the advantages in short running time. GA is better than ACS both in the quality of solutions and the running time of algorithm. In terms of the quality of solutions, the overall order in performance from high to low is SA, LDF, GA, ACS, LPT and LS.
